# Gastroprotective Effect of Hydroalcoholic Extract of Aloe *buettneri*

**Published:** 2011

**Authors:** Kossi Metowogo, Kwashie Eklu-Gadegbeku, Amégnona Agbonon, Kodjo A. Aklikokou, Messanvi Gbeassor

**Affiliations:** *Medicinal Plants Research Center, Faculty of Science, University of Loma, Togo*.

**Keywords:** *Aloe buettneri*, L-NAME, Indomethacin, L-arginine, Ulcer

## Abstract

*Aloe buettneri *A. Berger is commonly used in traditional Togolese medicine to treat inflammatory and gastric ulcers. The present study examined the gastro-protection effect of the hydro-alcoholic extract of *A. buettneri *on mucus production and gastric pH. A gastric ulcer is induced by ethanol 95° alone (1 mL/kg body weight), after pre-treatment with indomethacin (300 mg/kg) or by utilising L-NAME (40 mg/kg IV). In addition gastric mucus was removed by scraping and subsequently weighed. The experiment focused entirely on rats that had been subjected to fasting. The hydro-alcoholic extract of *A. buettneri *(500 mg/kg) significantly inhibited ulcers that were induced by ethanol, indomethacin or L-NAME pre-treatment. *A. buettneri *was shown to increase the production of gastric mucus. Furthermore L-arginine significantly decreased the size of the induced ulcers. The results achieved in the study carried out suggest that *A. buettneri *posses gastro-protective properties*.*

## Introduction

*Aloe buettneri *A. Berger (Lilliaceae) is a tropical cactus that is customarily used to control various diseases. These include chronic skin ulcers, coughs, dysmenorrhea, food poisoning, intestinal worms, difficult delivery, dysentery, general stomach aches, and lumbar pain (1). Other traditional uses for *A. buettneri *as reported by Togolese locals include antiseptic usage, purgative, decoagulant, larvicidal, vermifuge, stomachic tonic and as a stimulant. Telefo *et al*. (2, 3) reported that *A. buettneri *had beneficial effects on ovarian steroidogenesis.

Various species of Aloe including *A. vera *have been and are currently reportedly used to treat inflammation, ulcers, chronic wounds and others (4). Our previous work (5) showed that *A. buettneri*, a species related to *A. vera*, exhibited anti-ulcer and anti-inflammatory properties. These results are in line with those observed by Tan *et al*. (1) who evaluated the anti-ulcer and toxicity profile of *A. buettneri *in mice and Wistar rats. Available information was collected and then analysed, prompting interest to better understand the action mechanisms *A. vera *extract could provide elicit gastro-intestinal tract protection. 

Peptic ulcer is a major gastro-intestinal disorder caused by an imbalance between offensive (gastric acid, pepsinogen secretion...) and defensive (mucus secretion, gastric mucosal integrity.) factors (6). One mechanism utilised by anti-ulcer drugs is the reinforcement of gastric mucosa. Therefore, we suggest that the anti-ulcer effects exhibited by the hydro-alcoholic extract of *A.buettneri *may occur via gastro-protection. 

In the present work we will investigate the effect of *A. buettneri *extract on gastric mucus production and increases in pH.

## Experimental

*Plant material*

*A. buettneri *leaves were collected in April 2006 from the University of Lomé’s botanical garden located in the Faculty of Science department in Togo. These were then subsequently analysed and verified by the Laboratory of Botany. A reference sample was deposited in the Laboratory of Botany and Plant Ecology’s Herbarium, part of the Faculty of Science, “University of Lomé” (UL-MET 001)

*Extract preparation*

The selected leaves were washed, then dried under air-conditioning and finally ground to attain a powder. The powder was subsequently extracted with a mixture of water : ethanol (1:1, v/v) for 72 h and after that filtered. The filtrate was evaporated and a dark hydro-alcohol extract (yield: 24.25%) was obtained. Phytochemical screening of the extract revealed the presence of tannins, flavonoids and alkaloids.

*Animals*

Wistar rats, of both sexes weighing 150-200 g were the subject of testing. They were placed in an Animal house within the Faculty of Science’s Laboratory of Physiology/Pharmacology. The animals were kept under conditions of ambient temperature, humidity and dark-light cycle (12 h–12 h). The animals had free access to food and water. 

*Ethanol gastric ulcer induction*

A gastric ulcer was induced by an oral administration of 1 mL/100 g body weight of ethanol 95°. Rats were subjected to fasting for 24 h preceding their ulcer induction. The control group received distilled water and the treated groups received 250; 500 or 1000 mg/kg of *A. buettneri *extract (Ext) 30 min prior to ulcer induction. Lansoprazole (LPZ) 30 mg/kg was used as a reference drug. Two hours following ulcer induction the subjected rats were then sacrificed under ether anaesthesia. Their stomachs were removed and opened along the greater curvature. Following this the dimension of the ulcer was evaluated by means of planimetry, using 0.25 mm^2^ ulcer area by unit. The percentage of ulcer inhibition was then calculated using the following formula: (the dimension of the control rat’s ulcer-the dimension of the treated rat’s ulcer) the dimension of the control rat’s ulcer.

*Evaluation of gastric pH measurement*

Extract (250, 500 mg/kg; p.o.) was administered to rats and two hours later they were sacrificed under the same afore mentioned conditions. The stomach of each rat was removed, opened and the gastric pH was measured.

*Evaluation of mucus production*

Gastric mucus production was measured according to the method used by Salehi *et al*. (7). Two hours after the extract was administered (250, 500 mg/kg) the rats were sacrificed. Using a glass slide the gastric mucosa of each rat was gently scraped then, subsequently weighed using a precision electronic balance.

*Ethanol induced gastric mucosal lesion in L-NAME pre-treated rats*

The method used by Maria *et al*. (8) was implemented. Animals were subjected to fasting for 24 h and deprived of water for 19 h prior to commencing the experiment. The animals were divided into groups each consisting of 5 rats. Ethanol 95° was administered orally at a dose of 1 mL/100g body weight. Prior to ethanol administration, the rats orally received either distilled water or extract as follows:

Group I, control group, received saline solution (IV) and distilled water (orally), 45 and 30 min prior to ulcer induction.

Group II were administered saline solution (IV) and a 500 mg/kg extract of *A. buettneri *(orally), 45 and 30 min before ulcer induction.

Group III were given L-NAME 40 mg/kg (IV) and distilled water (orally), 45 and 30 min before ulcer induction. 

Groups IV and V received L-NAME 40 mg/kg (IV) and extracts of *A. buettneri *250 and 500 mg/kg (orally) 30 min before ethanol administration. 

Group VI was treated with L-arginine (IV) 400 mg/kg immediately, prior to being injected with L-NAME.

Two hours after the ulcers were induced, the rats were sacrificed. The stomach of each rat was removed, opened along the greater curvature and the dimension of the induced ulcer was evaluated by planimetry.

**Figure 1:**
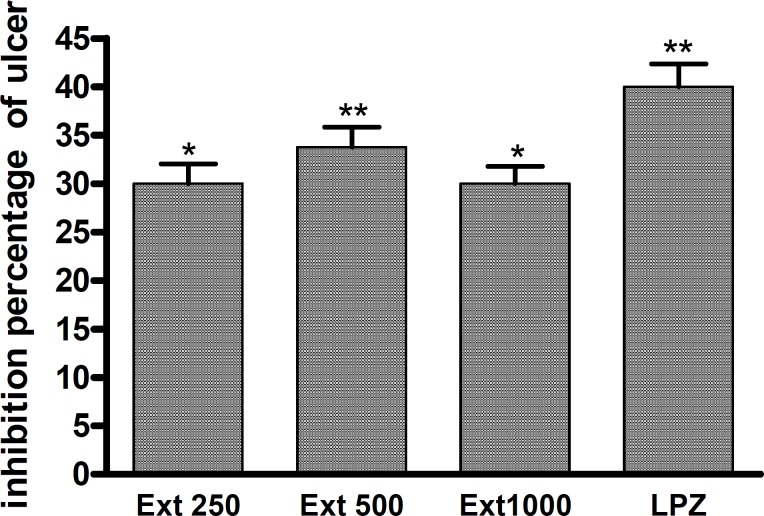
The effect of *A. Buettneri *hydro-alcohol extract on gastric mucosal damage induced by ethanol 95°. The extract was administered 30 min before ulcer induction. Results are mean ± SEM for 5 rats. *p < 0.05; **p < 0.01 (control vs treated).

*Ethanol induced gastric mucosal lesion in indomethacin pre-treated rats*

Ulcer inducement in indomethacin pre-treated rats was divided into three groups (I, III, and IV). Group I was used as a control (Co) and group II was not pre-treated with indomethacin instead receiving Nacl solution (IP). 30 min before indomethacin administration the rats were given distilled water or an extract of Indomethacin 300 mg/kg was administered (IP) to rats that had been subjected to fasting overnight. Four hours subsequent to the administration of indomethacin, the rats received ethanol 95° orally. Two hours later the rats were then sacrificed and treated as indicated above. 

*Statistical analysis*

Data obtained from the animal experiments was expressed as mean ± SEM statistical tests including a one-way analysis of variance (ANOVA) followed by bonferroni’s significant difference test were used to analyze any differences between the groups that were subjected to testing. A p-value of less than 0.05 was considered as being statistically significant.

## Results

The hydro-alcoholic extract of *A. buettneri *significantly inhibited (p < 0.05) ulcers induced by ethanol 95°. *A. buettneri *extract at doses of 250 mg/kg and 500 mg/kg led to inhibition percentages of 30 and 33.75% respectively. Contrastingly, when a dose of 1000 mg/kg was administered ulceration was reduced by approximately 30%.

**Figure 2:**
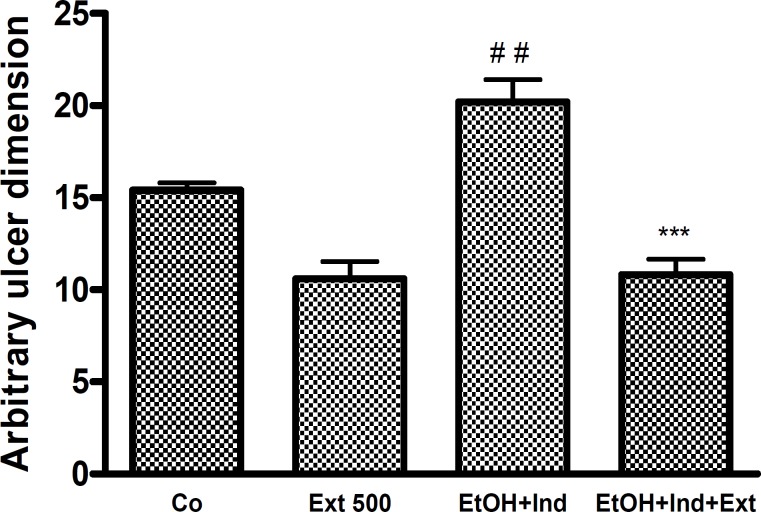
Effect of *A. buettneri *hydro-alcohol extract on the gastric pH. Extract was administered by gavage. Two hours after extract administration, cervical dislocation was used to kill the rats and their stomachs were kept. Gastric pH was measured using pH paper. Results are mean ± SEM for 5 rats. *p < 0.05 (Control vs treated)

The gastric pH of the control rats was 2.10 ± 0.24 versus 2.60 ± 0.24; 3.60 ± 0.40, and 3.10 ± 0.50 in rats treated with the *A. buettneri *extract at 250, 500 and 1000 mg/kg respectively (Figure 2). 

**Figure 3:**
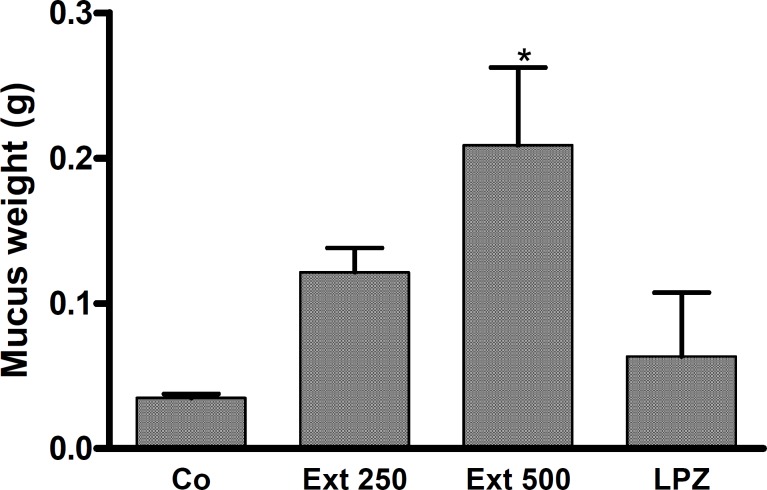
Effect of *A. buettneri *hydro-alcohol extract on gastric mucus production. Extract was administered by gavage. Two hours after ulcer induction rats were killed by cervical dislocation. Their stomachs were opened and mucus was removed by scraping. Results are mean ± SEM for 5 rats. *p < 0.05 (control vs treated).

The results of our study indicate that the extract of *A. buettneri *stimulates the production of gastric mucus. At doses of 500mg/kg the production of gastric mucus increased significantly (Figure 3).

**Figure 4:**
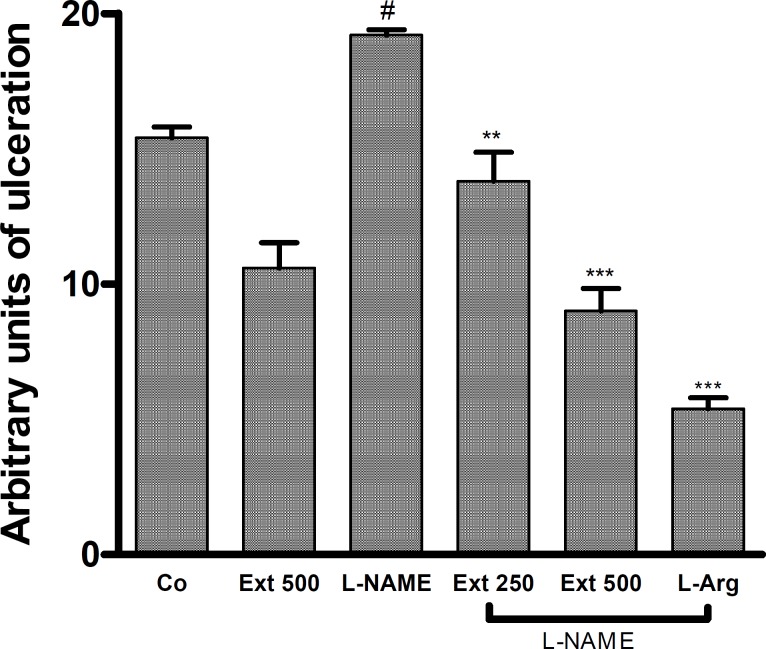
Effects of L-NAME (IV) and of combined treatment with L-Arginine (L-Arg) on the gastroprotective effect of *A. buettneri *hydro-alcohol extract (250; 500 mg/kg p.o.) against ethanol-induced gastric mucosal damage. Control group (Co) received distilled water. In all of groups the additional treatment was ethanol 95°. Ext = *A. buettneri *extract. The data is expressed as mean ± SEM for 5 rats. ***p < 0.001 (L-NAME vs L-NAME + Extract treated), ^#^ p < 0.05 (control vs L-NAME treated

The findings suggest that pre-treating animals with L-NAME (40 mg/kg, IV) exacerbated gastric damage induced by ethanol. The ethanolic extract of *A. buettneri *at 250 and 500mg/kg exerted significant protection against damage produced by ethanol (p < 0.01). Similar results were obtained for L-arginine, a NO donor, used as a positive control (Figure 4).

**Figure 5:**
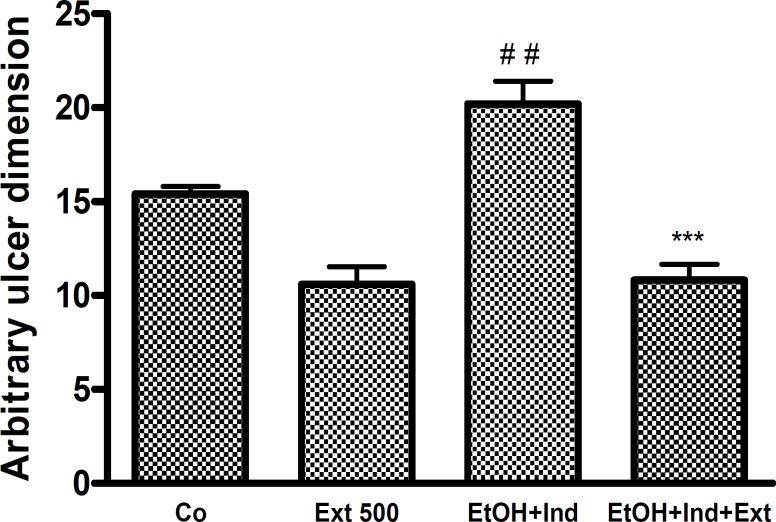
Effect of *A. Buettneri *hydro-alcohol extract on the gastric mucosal damage induced by indomethacin and ethanol 95°. Extract was administered 30 min before ulcer induction, indomethacin 300 mg/kg (IP) was given to the treated group and the control group (Co) received distilled water. After fours hour’s the rats received by gavage ethanol 95° at 1 mL/100g body weight. Results are mean ± SEM for 5 rats. **p < 0.01 (EtOH + Ind vs EtOH + Ind + Ext), ^##^ p < 0.01 (control vs EtOH + Ind).

Ulcers induced by ethanol in rats pre-treated with indomethacin were exacerbated. Significant differences were observed among groups treated with ethanol alone and indomethacin/ethanol. The extract significantly reduced the inducement of ulcers in rats pre-treated with indomethacin (Figure 5).

## Discussion

Gastric ulcers result from an imbalance involving gastric protection and aggressive factors (9). Gastric protection depends on a number of factors such as the release of prostaglandin E2 (PGE2), bicarbonate secretion, gastric mucus production and the regulation of gastric mucosal blood flow. Factors leading to gastric offensives are the hyper secretion of HCl or pepsin. Ethanol is well known to induce gastric ulcers via multi-factorial mechanisms such as the impairment of gastric defensive factors like mucus dissolution (10) or by increasing offensive factors such as acid secretion or gastrin release (11). 

*A. buettneri *extract inhibits ulcers induced by ethanol alone. Ethanol induced ulcers were exacerbated in rats pre-treated with indomethacin. Treatment with the extract 30 min before ulcers were induced, led to a significant decrease in ulcers induced by indomethacin/ethanol. The Inhibitory effect of the extract could as a result of prostaglandin stimulation. Indomethacin is known to inhibit cyclo-oxygenase 1 and 2 (COX-1 and COX-2), enzymes involved in prostaglandin synthesis. The shown protective actions exhibited against gastric injuries caused by ethanol are achieved through prostaglandin productionbyprostaglandin-producing enzymes (12). PGE_2_ synthesis has been shown to inhibit gastric ulcers by stimulating gastric mucus secretion (13) and by inhibiting gastric acid secretion. A previous study showed that a preparation of *A. vera *led to an increase in gastric pH by utilizing this mechanism (14). A related mechanism could be responsible for the effect exhibited by the extract of *A. buettneri *on gastric pH. 

The present study showed that *A. buettneri *extract increased gastric mucus production. Mucus is an important protective factor relating to gastric mucosa. According to Hiruma-Lima *et al*. (15) gastric mucus is a viscous, elastic, adherent and transparent gel formed by water and glycoproteins covering the entire gastrointestinal mucosa. These authors reported that the protective properties of the mucus barrier depends not only on its gel-like structure but are also related to the amount or thickness of the layer covering the mucosal surface. Mucus protects the gastric mucosa against irritants such as ethanol, HCl and acetyl acid. Magri *et al*. (16) reported that prostaglandin promotes mucus and bicarbonate secretions. It was demonstrated that PGE2 maintained gastric mucosal blood flow (17). Coruzzi *et al*. (18) showed that a lamtidine analogue with various NO-releasing moieties (furoxan, nitroxy and nitrosothiol) significantly inhibits ulcers induced by 0.6 N HCl. NO protects gastric mucosa by maintaining the integrity of gastric epithelium, regulating gastric mucosal blood flow and stimulating gastric mucus secretion and synthesis (19, 20). Furthermore Maria *et al*. (8) demonstrated that N^G^- nitro-L-arginine, a nitric oxide synthase (NOS) inhibitor, exacerbates ulcers induced by ethanol as the protective effect of dehydroleucodine (NO production stimulator) was counteracted by pre-treating with L-NNA. The *A. buettneri *extract inhibits ulcers induced by ethanol in rats pre-treated with N^G^-nitro-L-arginine methyl ester (L-NAME), an analogue of L-NNA. 

The extracts effect of increasing gastric mucus production could be mediated by endothelium NO production.

We conclude that the hydro-alcoholic extract of *A. buettneri *has a gastro-protective effect on the gastric tract. The extract increased mucus production and gastric pH. Further investigations are required to fully understand the action mechanisms of the extract. 
